# CEF3 is involved in membrane trafficking and essential for secondary cell wall biosynthesis and its mutation enhanced biomass enzymatic saccharification in rice

**DOI:** 10.1186/s13068-022-02205-y

**Published:** 2022-10-14

**Authors:** Hongrui Jiang, Yan Ren, Junyao Guo, Huijie Yang, Xiaotong Zhu, Wenhao Li, Liangzhi Tao, Yue Zhan, Qi Wang, Yuejin Wu, Binmei Liu, Yafeng Ye

**Affiliations:** 1 Key Laboratory of High Magnetic Field and Ion Beam Physical Biology, Hefei Institutes of Physical Science, Chinese Academy of Sciences, Hefei, 230031 China; 2 Anhui Province Key Laboratory of Environmental Toxicology and Pollution Control Technology, Hefei Institutes of Physical Science, Chinese Academy of Sciences, Hefei, 230031 Anhui China

**Keywords:** Secondary cell wall (SCW), Rice, Cellulose biosynthesis, Biomass, Saccharification, membrane trafficking, Map-based cloning

## Abstract

**Background:**

As one of the most important staple food crops, rice produces large of agronomic biomass residues that contain lots of secondary cell walls (SCWs). Membrane trafficking plays key roles in SCWs biosynthesis, but information association membrane trafficking and SCWs formation in plants is limited.

**Results:**

In this study, we report the function characterization of a rice mutant, *culm easily fragile* 3 (*cef3*), that exhibits growth retardation and fragile culm phenotype with significantly altered cell wall composition and reduced secondary wall thickness. Map-based cloning revealed that *CEF3* encodes a homologous protein of Arabidopsis STOMATAL CYTOKINESIS DEFECTIVE2 (SCD2). The saccharification assays revealed that CEF3 mutation can improve biomass enzymatic saccharification. Expression pattern analysis indicated that *CEF3* is ubiquitously expressed in many organs at different developmental stages. Subcellular localization revealed that CEF3 is a Golgi-localized protein. The FM4-64 uptake assay revealed CEF3 is involved in endocytosis. Furthermore, mutation of *CEF3* not only affected cellulose synthesis-related genes expression, but also altered the abundance of cellulose synthase catalytic subunit 9 (OsCESA9) in the PM and in the endomembrane systems.

**Conclusions:**

This study has demonstrated that CEF3 participates in the membrane trafficking that is essential for normal cellulose and other polysaccharides biosynthesis of the secondary cell wall, thereby manipulation of *CEF3* could alter cellulose content and enhance biomass enzymatic saccharification in rice plants. Therefore, the study of the function of *CEF3* can provide a strategy for genetic modification of SCWs in bioenergy crops.

**Supplementary Information:**

The online version contains supplementary material available at 10.1186/s13068-022-02205-y.

## Background

The plant cell wall is a basic cellular structure that not only plays important role in plant growth and development, but also represents one of the most plentiful natural resources on earth [1]. Plants produce two typical types of cell walls, the primary cell walls (PCW) surrounding in all cells and the secondary cell walls (SCW) which are rigid and thickened structures in specific cell types [1]. The secondary cell walls not only provide mechanical strength for plant body, but also greatly contribute to the bulk of renewable plant biomass [2]. Rice is one of the most important staple crops for feeding half of population in the world [3]. Rice plants mechanical strength directly associate with lodging and ultimate yield. In addition, rice straw is a very important raw material for the production of bioenergy and bio-based products [4]. However, due to the lignocellulose recalcitrance of the secondary cell wall, the utilization of rice straw for bio-energy is very limited [5, 6]. Hence, to understand the mechanism of SCWs biosynthesis can provide a strategy for improving rice lodging resistance and/or manipulating plant biomass production. The SCWs are typically composed of cellulose, hemi-cellulose and lignin [7].

In the past decades, many studies for secondary cell walls biosynthesis have been reported in both dicot and monocot plants [8]. In current understanding, the cellulose is synthesized at the plasma membrane (PM) by the cellulose synthase complex (CSC) containing at least three different cellulose synthases, which are assembled into CSCs in either the endoplasmic reticulum (ER) or the Golgi apparatus and trafficked by vesicles to the plasma membrane (PM) [9]. Nevertheless, the hemi-cellulose and lignin are produced in the Golgi apparatus, and are then transported to the walls through secretory pathways [10]. Therefore, vesicular trafficking of required proteins and SCW materials from the Golgi apparatus to the PM or the extracellular matrix plays a very important role for SCW biosynthesis. Decadal researches have discovered several factors involved in vesicular trafficking of proteins and polysaccharides in the cell wall [11–13]. Live-cell imaging of fluorescently tagged CESAs has provided direct evidence of CSC trafficking [14]. The CSCs have been detected in the Golgi apparatus, and the glycosyltransferases STELLO (STL1 and STL2) are involved in regulating CSCs assembly in the Golgi [15]. CSCs are then trafficked to the PM through the *trans*-Golgi network/early endosome (TGN/EE) compartment [16]. Membrane trafficking depends on intermediate compartments and a large number of proteins for ensuring accuracy and directionality of each transport route [12]. The CESAs are known to localize in two types of compartments, the vacuolar-type H^+^-ATPase subunit a1 (VHA-a1)/SYP61 compartment and the microtubule-associated cellulose synthase compartment (MASC) [17]. Although the membrane trafficking mechanism of enzymes involved in cell wall biosynthesis is relatively clear, little is known about the membrane trafficking of polysaccharide. This is partially attributed to technical challenges in biochemically determining polysaccharide cargo in specific vesicles [11]. Recent research revealed SYP61 vesicles are involved in the transport and deposition of structural polysaccharides and glycoproteins [11].

Clathrin-coated vesicle (CCV) is surrounded by clathrin lattice formed at PM and TGN/EEs in plant cells and plays important roles in vesicular trafficking [18]. The clathrin-mediated trafficking pathways are regulated by trafficking factors including Rab and ADP-ribosylation factor (ARF)-related small GTPases [19, 20], and soluble N-ethylmaleimide-sensitive factor adaptor protein receptors (SNARE) proteins [21]. Arabidopsis *stomatal cytokinesis defective1* (*scd1*) and *scd2* mutants exhibit dwarfism and have defects in cell division and expansion phenotypes, which are similar to exocyst mutants [22, 23]. SCD1 and SCD2 together constitute the SCD complex that cooperates with members of the exocyst complex and RabE1 GTPases to mediate post-Golgi trafficking to the PM [22]. In addition, dynamin-related proteins (DRPs) are essential for CCV formation through scission of the budding vesicle from the PM [24]. In rice, *BC3*, encoding a OsDRP2B participates in the endocytic pathway, probably as well as in post-Golgi membrane trafficking. Mutation of BC3/OsDRP2B disturbs the membrane trafficking that is essential for normal cellulose biosynthesis of the secondary cell wall, thereby leading to inferior mechanical properties in rice plants [13]. Clathrin-mediated vesicle trafficking is a well-studied transport pathway in plant growth and development [25–28], but how it regulates SCW biosynthesis remains elusive.

In this study, we identified a novel rice *culm easily fragile* 3 (*cef3*) mutant, that exhibits growth retardation and fragile culm phenotype with significantly altered cell wall composition and reduced secondary wall thickness. Map-based cloning revealed that *CEF3* encodes a homologous protein of Arabidopsis STOMATAL CYTOKINESIS DEFECTIVE2 (SCD2). We further revealed mutation of *CEF3* not only affected cellulose synthesis and hemicellulose synthesis-related genes expression, but also altered the abundance of cellulose synthase catalytic subunit 9 (OsCESA9) in the PM and in the endomembrane systems. The saccharification assays revealed that CEF3 mutation can improve biomass enzymatic saccharification. Based on these findings, we can conclude that CEF3 participates in the membrane trafficking that is essential for normal cellulose and other polysaccharides biosynthesis of the secondary cell wall, thereby manipulation of *CEF3* could alter cellulose content and enhance biomass enzymatic saccharification in rice plants.

## Results

### The *cef3* mutant results in altered mechanical strength and growth status

**Fig. 1:**
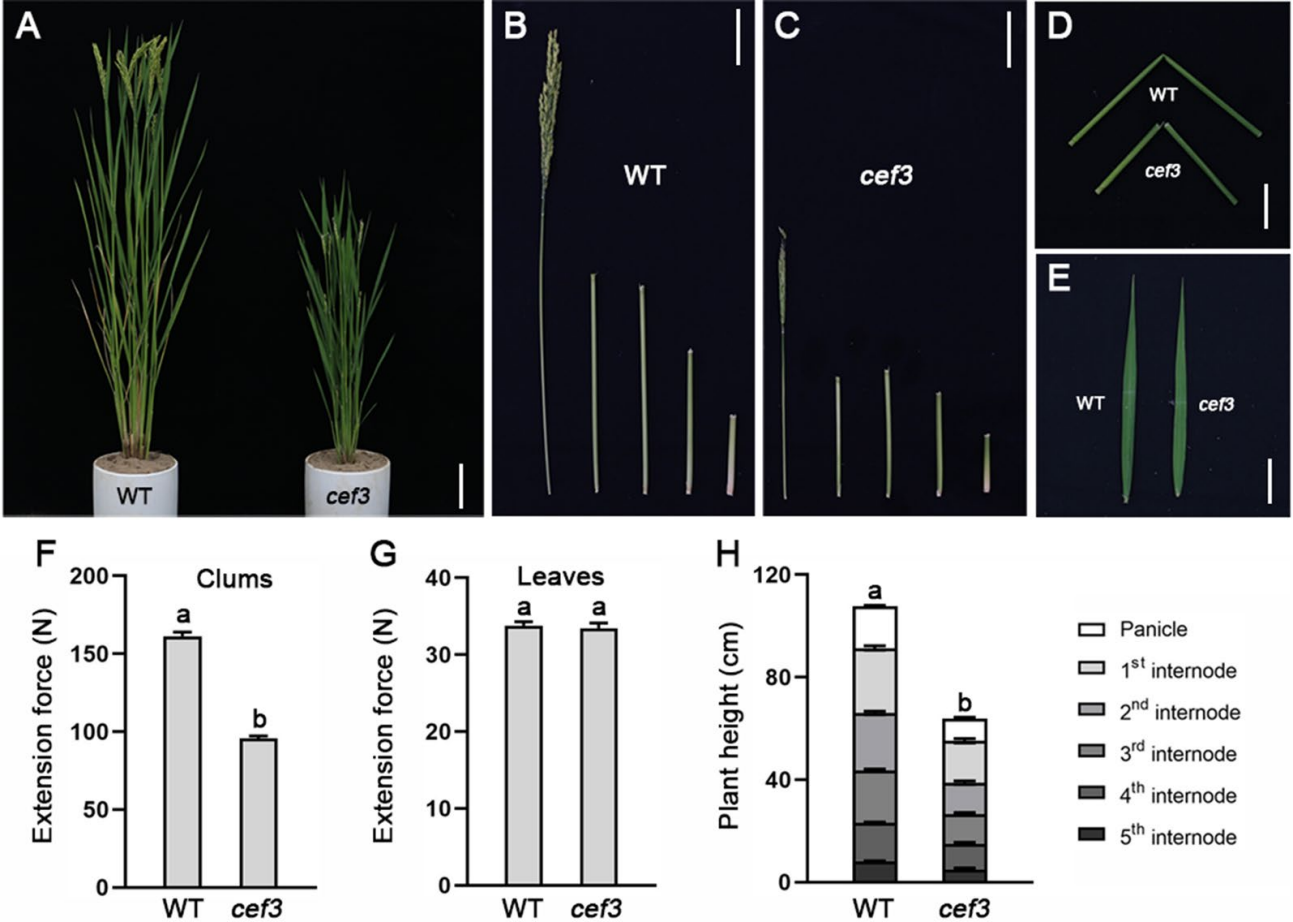
Phenotypic comparison between the wild-type and *cef3* plants. **A** Phenotype of the wild-type (WT) and *cef3* plants at the mature stage. Scale bars: 10 cm. **B**, **C** Comparison of the internode lengths between the wild-type and *cef3* culms. Scale bar: 5 cm. **D**, **E** Brittleness of culms and leaves. Scale bars: 5 cm. **F**, **G** Measurements of the extension force of culms and leaves. **H** Plant height containing each internode length. Error bars represent SE (*n* = 30). Different letters denote significant differences (*P* < 0.05, Duncan’s multiple range test)

The *culm easily fragile 3* (*cef3*) mutant was obtained from the *japonica* cultivar, Xiushui63 by treatment with a heavy ion beam. Compared to wild type (WT), we observed that the *cef3* mutant had obviously reduced plant height, due to small panicle and shorten internodes (Fig. 1A, B, C, H) . In addition, the *cef3* mutant exhibited easily broken culms but not leaves (Fig. 1D, E). Unlike most other brittle culm mutants previously reported [4, 29–36], the *cef3* mutant showed no easily broken phenotype at seedling stage (Additional file 1: Figure S1). To accurately describe the broken phenotype, we quantitatively compared the extension forces of the second internodes and the flag leaves in the WT and *cef3* plants. The results indicated the force applied to break *cef3* internodes was reduced by more than 40% compared with that of the wild type, but no significant difference in leaves (Fig. 1F, G).

**Fig. 2:**
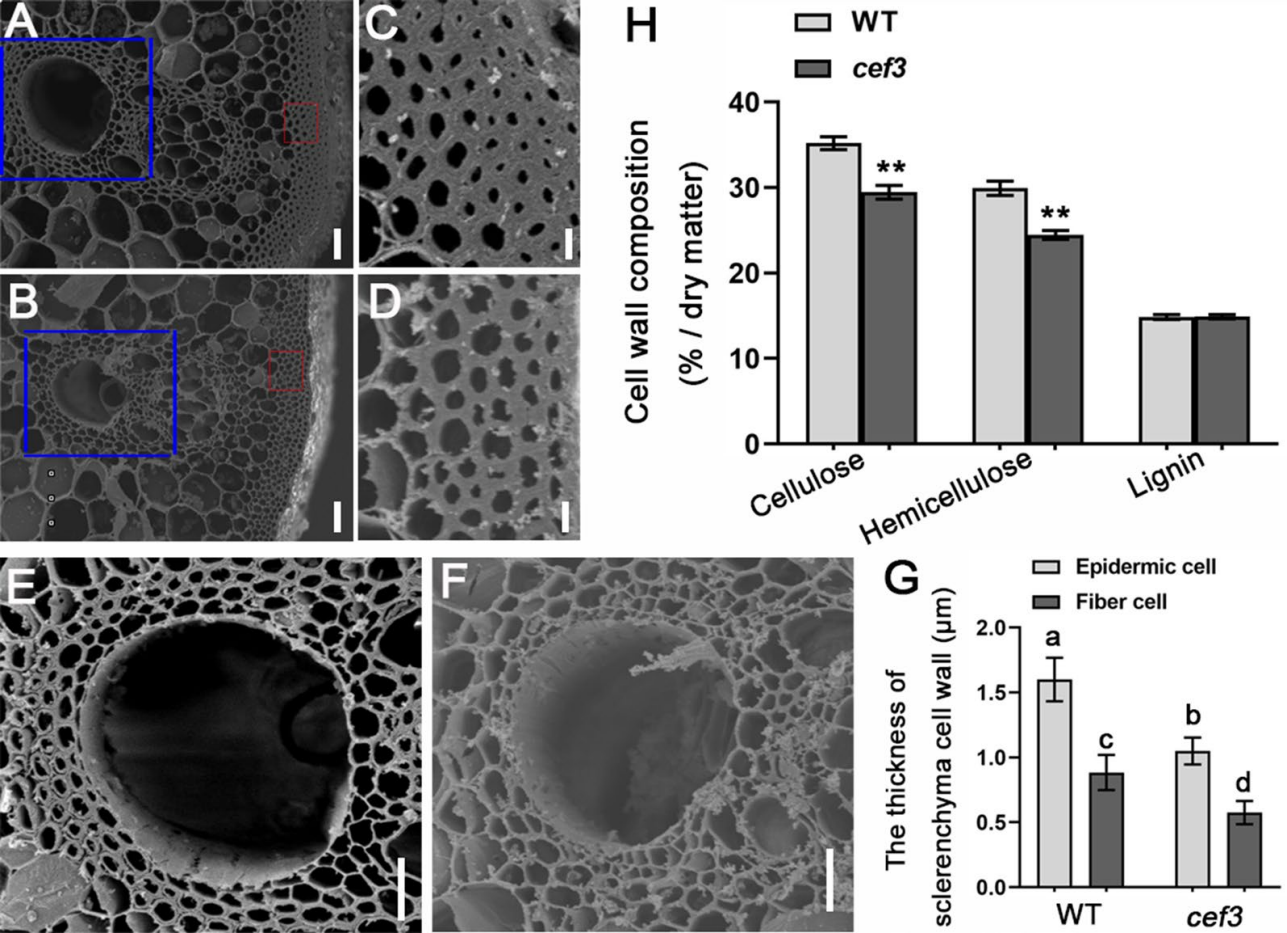
Observations of stem tissues and cell wall structures. **A**, **B** Scanning electron micrographs of the sclerenchyma cell walls of the wild-type (A) and *cef3* (B), Bars = 100 μm. **C**, **D** Enlargements of the red boxed regions in (A) and (B), respectively. Bars = 5 μm. **E**, **F** Enlargements of the blue boxed regions in (A) and (B), respectively. Bars = 20 μm. **G** Measurements of the thickness of sclerenchyma cell wall. Error bars represent SD (*n* = 30). Different letters denote significant differences (*P* < 0.05, Duncan’s multiple range test). **H** Cell wall polymer determination. Error bars, SD of three biological replicates. The asterisks (**) indicate p < 0.01 from Student’s *t* tests

Reduced thickness of the sclerenchyma cell walls is the main reason why rice plants shown brittle culm phenotype. Therefore, we used scanning electron microscope (SEM) to observe the second internodes cross sections of WT and *cef3* plants. The observations revealed that the sclerenchyma cell wall of *cef3* was extremely thinned (Fig. 2B, D, F, G), compare with the WT (Fig. 2A, C, E, G).

The *cef3* defects in mechanical strength and wall structure suggested that the cell wall composition in the mutant plants may be altered. Therefore, we examined the cell wall composition in the second internodes of the WT and *cef3* plants at the mature stage. As shown in Table 1, the cellulose content was significantly deceased in *cef3* internodes, whereas the neutral sugars content derived from non-cellulosic polysaccharides were generally increased in *cef3* samples, except for the xylose (Xyl), which is the major sugar of hemicellulose. We also detected the hemicellulose content was significantly decreased in *cef3* internodes, but the lignin content has no significant change, compared to WT (Fig. 2H).

**Table 1:** Cell wall composition analysis of internodes of wild type, *cef3* and *pCEF3F* plants

Sample	Rha	Fuc	Ara	Xyl	Man	Gal	Glu	Cellulose
Wild type	1.67 ± 0.07	0.61 ± 0.02	18.66 ± 1.37	139.93 ± 4.98	1.05 ± 0.11	6.46 ± 1.04	51.70 ± 8.02	455.25 ± 11.61
*cef3*	1.97 ± 0.06^a^	0.80 ± 0.05	24.92 ± 1.23^a^	114.23 ± 5.71^a^	1.17 ± 0.14	15.66 ± 0.58^a^	102.21 ± 0.60^a^	385.63 ± 23.68^a^
*pCEF3F*	1.66 ± 0.06	0.65 ± 0.03	18.74 ± 0.86	135.35 ± 5.68	1.09 ± 0.21	6.57 ± 0.39	54.32 ± 1.72	468.32 ± 9.68

The results are means ± SE of five independent assays. Each wall component was calculated as mg·g^−1^ of alcohol-insoluble cell-wall residue

^a^Significant difference (*t* test at *P* < 0.01) with respect to wild-type

Taken together, the *cef3* defects in mechanical strength was correlated with the thinned sclerenchyma cell walls and complicated alterations in wall composition.

### Map-based cloning of *CEF3*

**Fig. 3:**
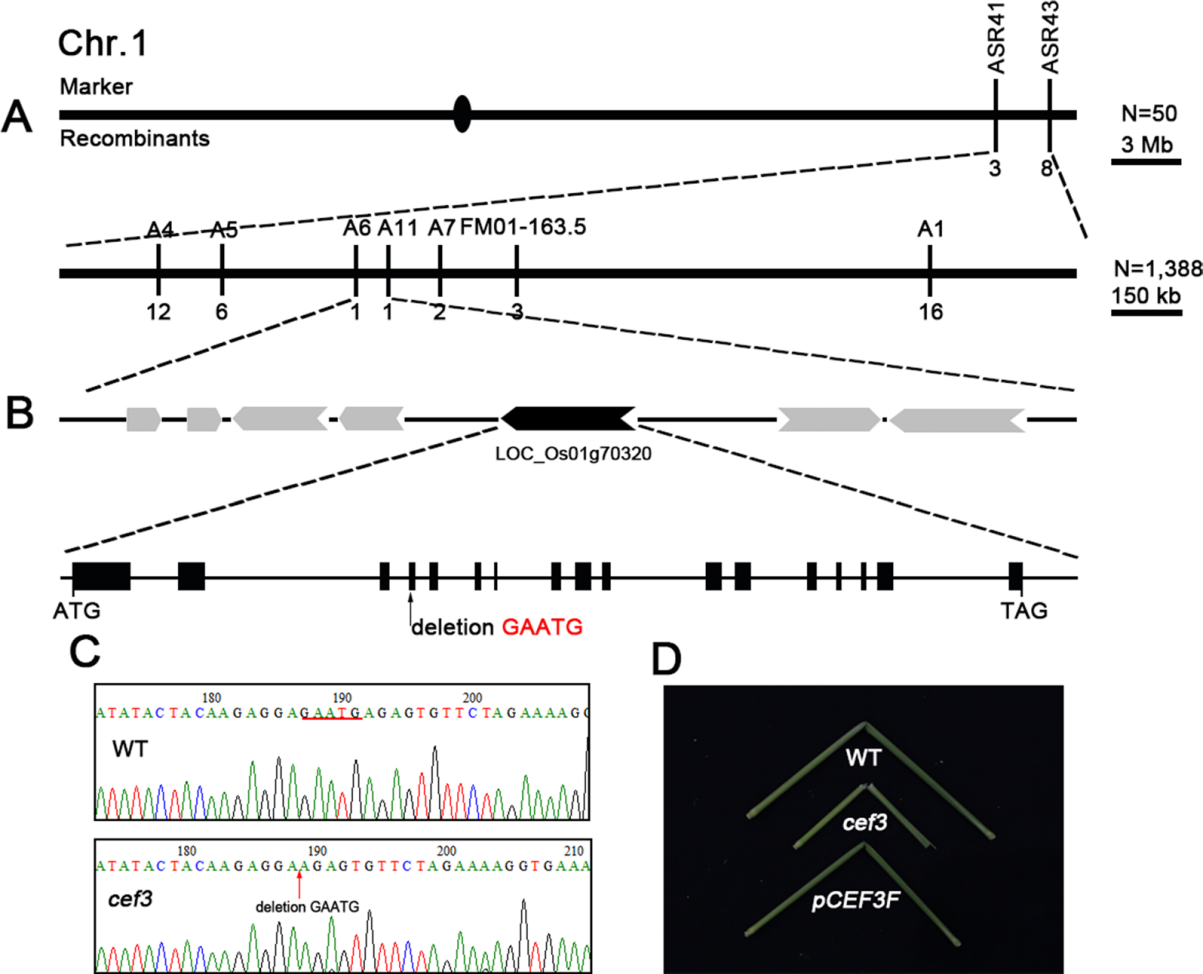
Map-based cloning of the CEF3 gene. **A** *cef3* locus was mapped between molecular markers A6 and A11 in a 52-kb region in chromosome 1. **B** 7 predicted ORFs within the fine mapping region and sequencing analysis revealed a 5-bp deletion in the fourth exon of LOC_Os01g70320. **C** DNA sequence alignments of wild-type and *cef3* mutant. **D** Folding the internodes of rice plants to show the rescued mechanical property in the complemented plants

To understand the molecular basis of the above phenotypes, a map-based cloning approach was performed to isolate the *CEF3* gene. We crossed the mutant with 93–11, a wild type polymorphic *indica* variety, to generate a F_2_ mapping population. The *cef3* locus was located between molecular markers ASR41 and ASR43 on chromosome 1, and further narrowed within an approximately 52-kb region between markers A6 and A11 (Fig. 3A). The 52-kb region contains 7 putative open-reading frames (ORFs) (Fig. 3B). Then, we sequenced all of the ORFs and a 5-bp deletion was found in LOC_Os01g70320 (Fig. 3B, C). This deletion occurred in the fourth exon of the ORF, resulting a frame-shift and premature translational product of 235 amino acids (Fig. 3B and Additional file 2: Figure S2). Therefore, *cef3* is very likely to be a loss-of-function mutation. To confirm that LOC_Os01g70320 corresponds to the *cef3* locus, a 4.2-kb DNA fragment containing the 2.5-kb putative promoter and the coding region was cloned vector *pCAMBIA2300* to generate the plasmid *pCEF3::CEF3* (*pCEF3F*), which was introduced into the *cef3* plants by Agrobacterium tumefaciens-mediated transformation. The transgenic *cef3*^*pCEF3::CEF3*^ plants complete rescued the dwarfism (Additional file 3: Figure S3), fragile culm phenotype and the altered cell wall composition (Fig. 3D and Table 1).

**Fig. 4:**
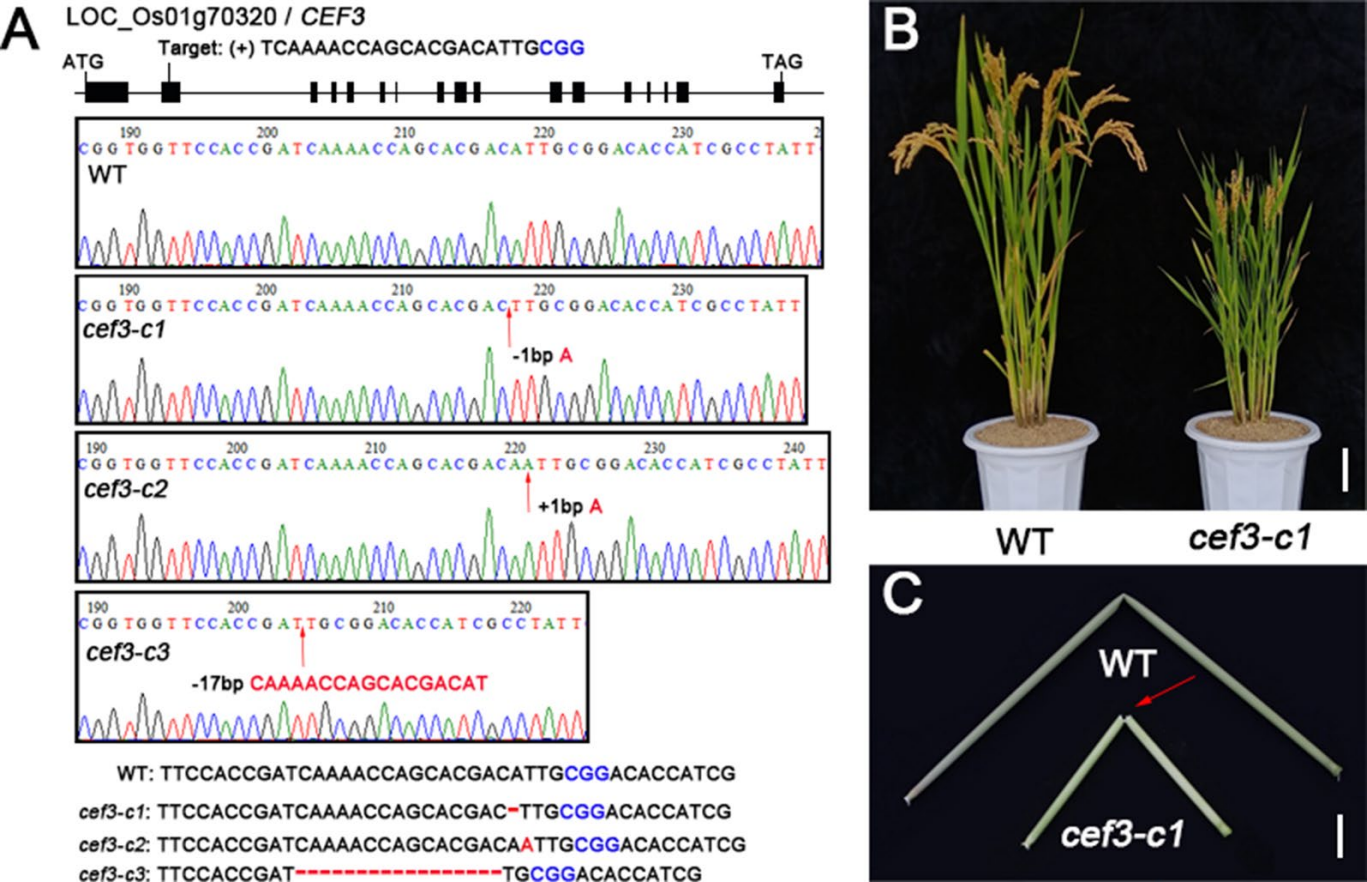
Generation and analysis of *cef3* mutants. **A** DNA sequence alignments for the three homozygous *cef3* mutants identified in the T1 generation, together with a wild-type (WT) control. **B** Phenotype of the wild-type (WT) and *cef3-c1* plants at the mature stage. Scale bars: 10 cm. **C** Mature culms of wild-type and *cef3-c1* after hand flexing. Scale bars: 1 cm

To further verify the function of *CEF3*, the CRISPR/Cas9 approach was performed to generate other mutant alleles of *CEF3* in wuyunjing7 (W7) background. We designed a sequence-specific single guide RNA (sgRNA) target site (TCAAAACCAGCACGACATTG), which in the second exon of *CEF3*. Three transgene-free homozygous knockout lines with different genotypes, *cef3-c1*, *cef3-c2* and *cef3-c3* were obtained (Fig. 4A). Protein sequence alignments of the three homozygous mutants and the wild type protein revealed that *cef3-c1*, *cef3-c2* and *cef3-c3* showed coding frame shifts and premature translational stops (Additional file 4: Figure S4). All of these mutants exhibited dramatically dwarfism (Fig. 4B; Additional file 6: Figure S6A, C) and culms easily broken phenotype (Fig. 4C; Additional file 6: Figure S6B, D). We also found that the cell wall compositions were altered in *cef3-c1* plants, compare with its wild type (Additional file 9: Table S1). Therefore, the LOC_Os01g70320 was the *CEF3* gene responsible for the mutant phenotypes described above.

### The *cef3* mutant enhances biomass saccharification

**Fig. 5:**
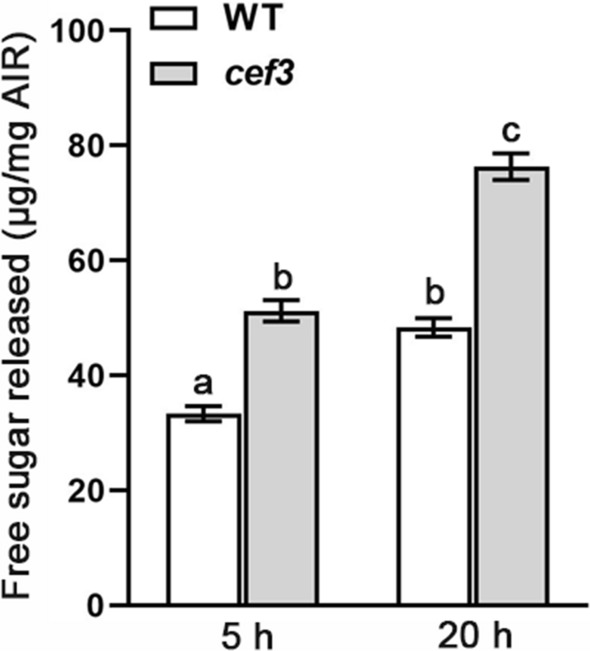
Saccharification analysis of the wall residues from WT and *cef3* internodes. The wall residues were treated with enzyme mixture for 5 and 20 h. Error bars indicate SE from the mean of three replicates. Different letters denote significant differences (*P* < 0.05, Duncan’s multiple range test)

In recent years, rice straw has been highlighted as an important material for biofuel production, but the lignocellulose recalcitrance determined by high cellulose content and crystallinity directly lead to costly biomass processing. As previously reported, the lower cellulose content rice mutants can improve enzymatic saccharification [29, 31, 32]. To detected whether the *CEF3* mutation also enhances biomass saccharification. We examined the saccharification efficiency of lignocellulosic material derived from WT and *cef3* plants. The sugar yields were significantly higher in *cef3* than that of WT (Fig. 5). To further prove CEF3 mutation can improve enzymatic saccharification, we also examined the saccharification efficiency of lignocellulosic material derived from *cef3-c1* plants generated by CRISPR-Cas9 system. The sugar yields were also significantly higher in *cef3-c1* than that of WT (Additional file 5: Figure S5). These results suggest that the *CEF3* mutation can enhance biomass enzymatic saccharification.

### *CEF3* is ubiquitously expressed in many organs at different developmental stages

**Fig. 6:**
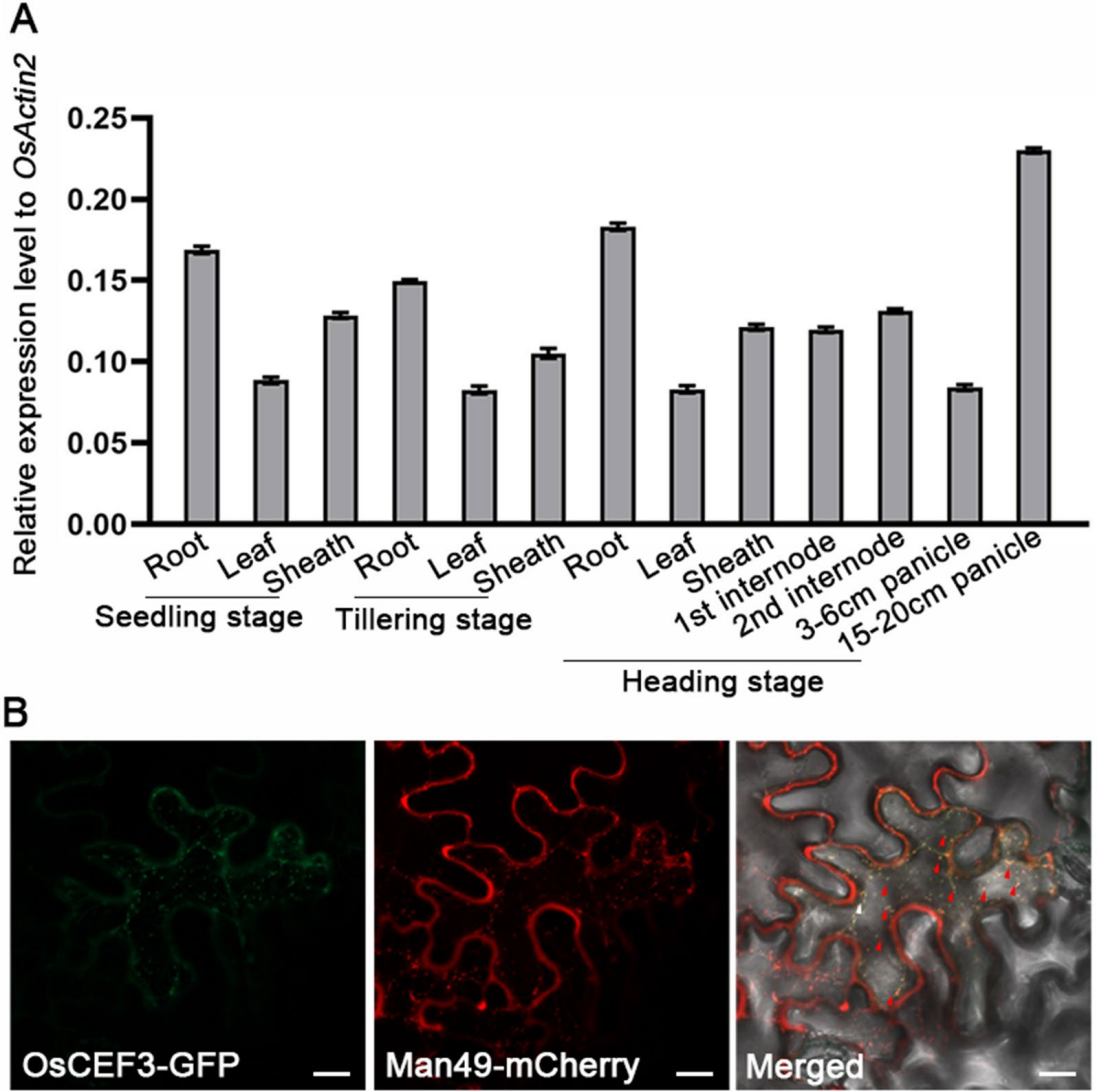
Expression pattern and subcellular localization of CEF3. **A** qRT-PCR analysis of *CEF3* expression in various rice organs and different developmental stage, using the *OsActin2* as an internal control. **B** Full-length CEF3 fused with green fluorescent protein (GFP) and transfected into tobacco leaves together with Man49-mCherry. Bars = 20 μm. The red arrows indicate colocalization signals at the Golgi

To characterize the spatial and temporal expression profile of *CEF3*, we performed quantitative real-time (qRT)-PCR to analyze the expression level of *CEF3* in various tissues at different developmental stages. The results suggested that *CEF3* is ubiquitously expressed in all organs, with relatively high levels in roots of different developmental stages and 15–20 cm length panicles. However, the expression level of *CEF3* is relatively low in leaves at any developmental stages (Fig. 6A). This expression pattern fits with the pleiotropy phenotypes (dwarfism, culm brittleness, small panicle etc*.*) of *cef3*.

### CEF3 is a plant-specific protein homologous to AtSCD2

The coding sequence of the *CEF3* is 1713 bp in length and encodes a protein of 570 amino acid residues with a predicted molecular mass of approximately 63 kDa. A BLASTP search for CEF3 homologs in Oryza sativa (*japonica*) and Arabidopsis thaliana genomes identified 2 and 5 closer homologs, respectively (Additional file 7: Figure S7A). Phylogenetic analysis revealed that CEF3 is a homolog of AtSCD2 (At3g48860), which functionally interacts with subunits of the exocyst complex for regulating proper membrane trafficking. CEF3 and AtSCD2 shared three conserved coiled-coil (CC) domains in the mid-region and a SCD2 domain with unknown function at the C terminus (Additional file 7: Figure S7B). The subcellular distribution of proteins often provides important clues for understanding their cellular functions. To explore the subcellular localization of CEF3, we fused CEF3 with a green fluorescence protein (GFP) and cotransfected this chimeric construct in *Nicotiana benthamiana* leaves with a mCherry fused Golgi marker, Man49. The overlapping signals indicated that CEF3 is a Golgi-localized protein (Fig. 6B).

**Fig. 7:**
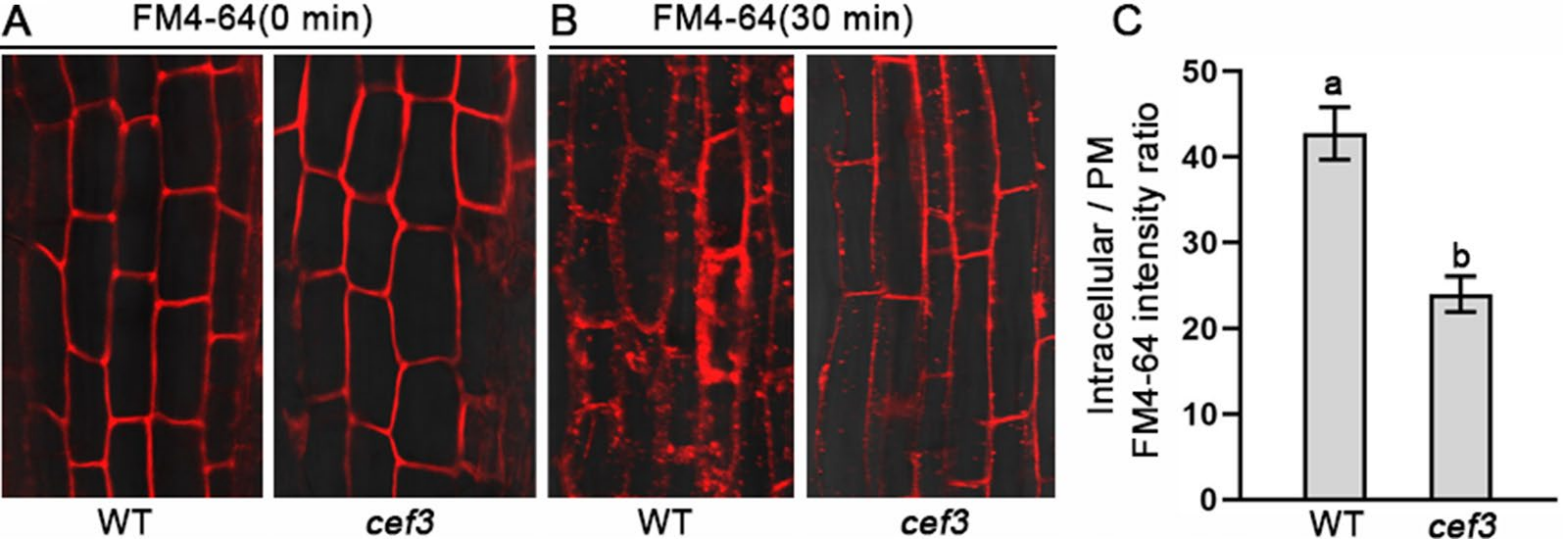
CEF3 is involved in endocytic pathway. **A**, **B** Confocal microscopy images showing the internalization of FM4–64 in root cells of WT and *cef3* at 0 min (A) and 30 min (B), FM4-64 signals within 0 min (A) and 30 min (B) of treatment. The *cef3* mutant reduces the internalization of FM4-64. **C** Quantitative analyses of FM4–64 fluorescence intensity ratio of intracellular and PM in (B) (*n* = 30). Error bars indicate SE from the mean of three replicates. Different letters denote significant differences (*P* < 0.05, Duncan’s multiple range test)

As previously reported [23], AtSCD2 is required for post-Golgi and/or endocytic trafficking and given that CEF3 is a Golgi-localized protein, we hypothesized that it might also has a function in membrane trafficking. To test this hypothesis, we used confocal laser scanning microscope to monitor the internalization levels of FM4-64 in root cells of WT and *cef3* seedlings. The results indicated that accumulation of FM4-64 in the *cef3* mutant was lower than in the WT (Fig. 7A, B). Quantification of FM4-64 internalization assay revealed that the relative internalization of FM4-64 was significantly decreased in *cef3* mutant roots compared with its WT (Fig. 7C). All above results indicated that CEF3 participated in membrane trafficking, similar with its homolog of AtSCD2.

### CEF3 regulates secondary cell wall-related gene expression and OsCESA9 abundance at the PM

**Fig. 8:**
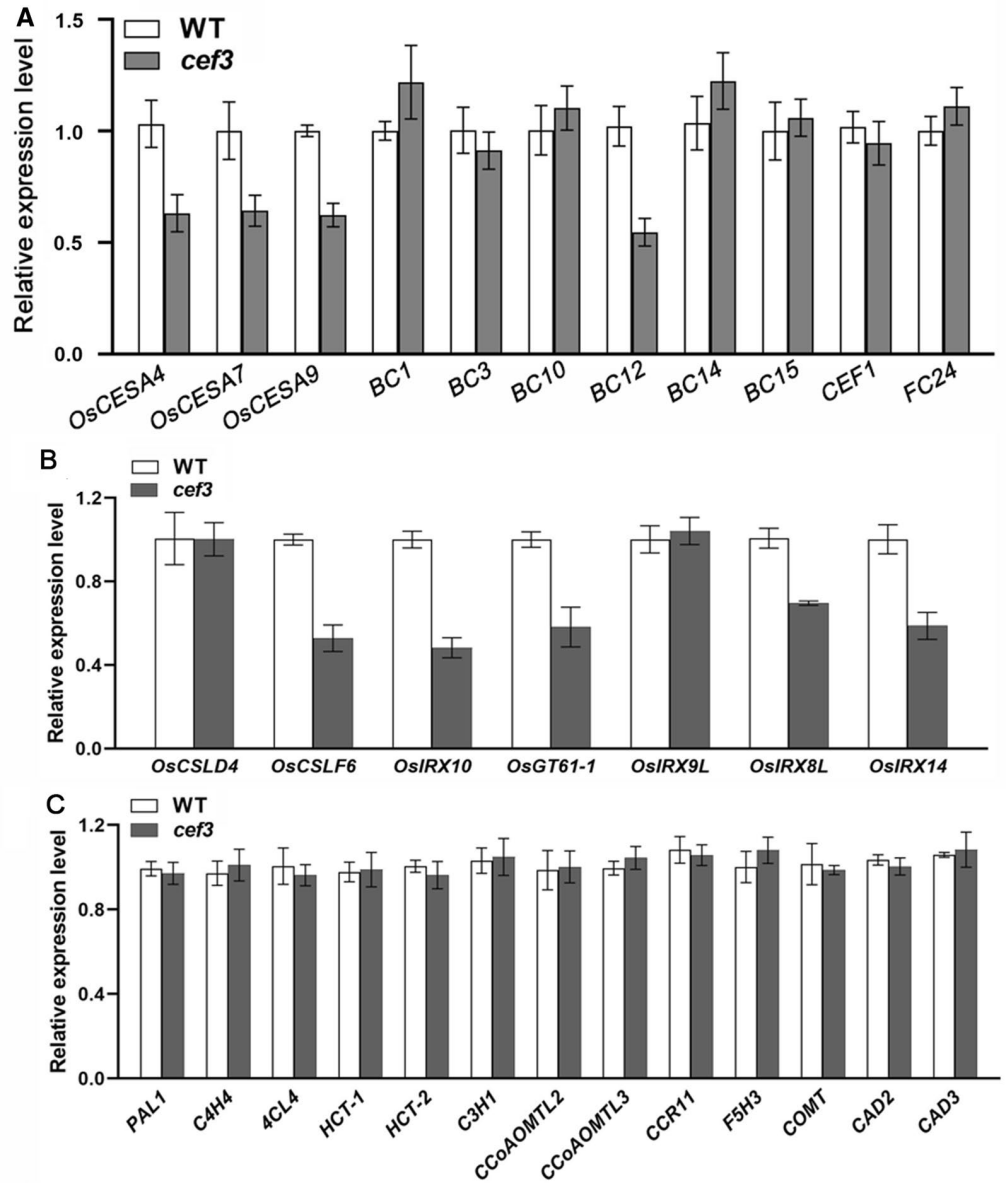
Expression analysis of cell wall biosynthesis-related genes. **A** Relative expression levels of *BC* genes. **B** Relative expression levels of xylan biosynthesis genes. **C** Relative expression levels of lignin biosynthesis genes. *OsActin2* was used as the internal control and data from three independent biological replicates were analyzed

**Fig. 9:**
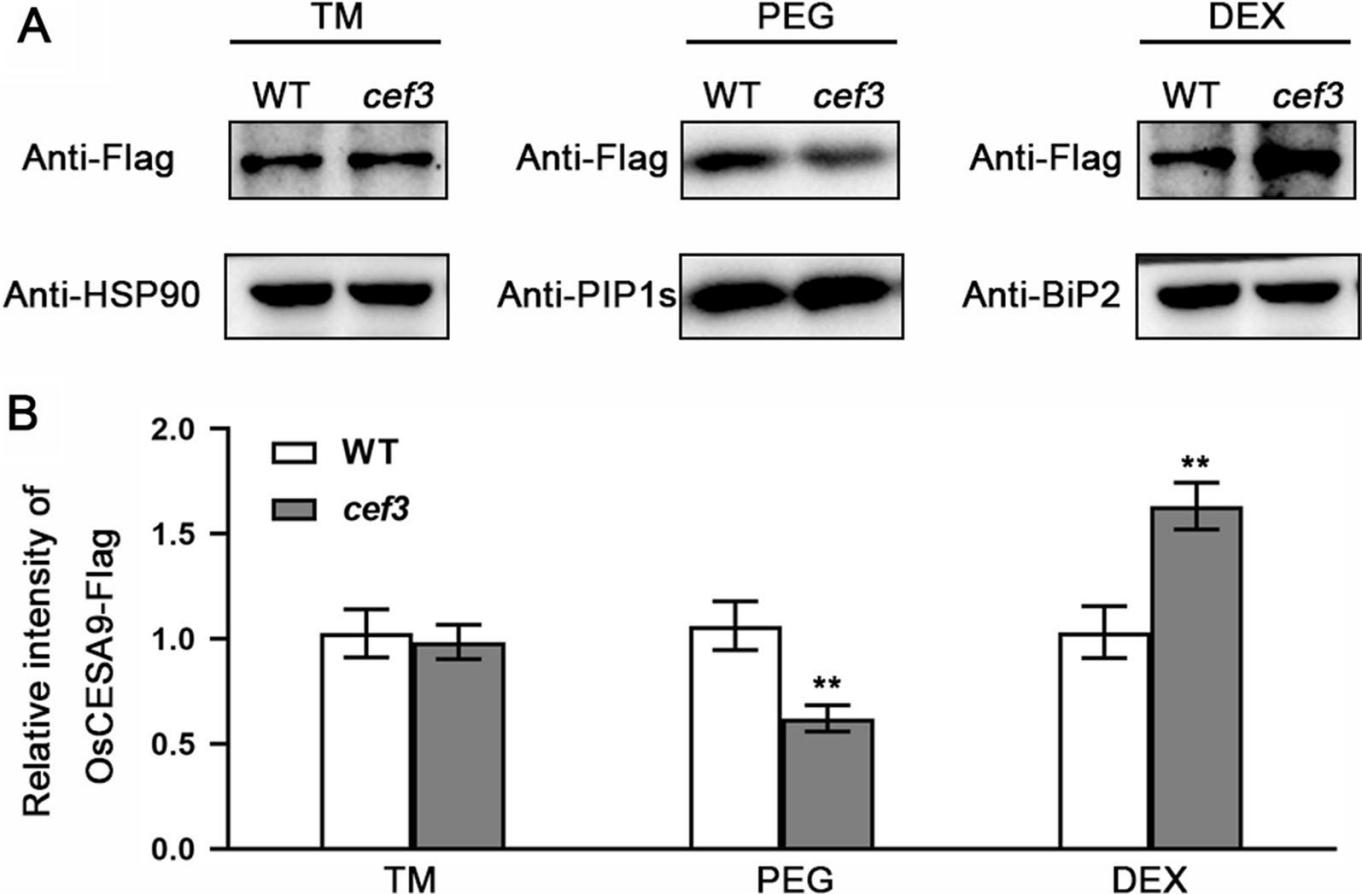
cef3 mutation affects the abundance of OsCESA9 at the plasma membrane. **A** Western blotting of different fractions of OsCESA9 proteins with anti-Flag antibodies. TM, total membranes; PEG, the plasma membrane fraction; DEX, the endomembrane fraction; Anti-HSP90, internal control; Anti-BiP2 and Anti-PIP1s antibodies are used to label the marker protein in the endomembrane and plasma membrane, respectively. Western blotting analysis has been repeated for at least 3 times. **B** Quantification of the relative chemiluminescent signal intensity shown in (A). Intensity detected in wild-type plants is set to 1 (means of three biological replicates). Error bars indicate SE from the mean of three replicates. ** indicate *P* < 0.01 (*t* test, compared with the WT value)

To explain the reason why *cef3* mutant exhibits brittle culm phenotype and alters cell wall composition. We first detected the secondary cell wall-related genes expression in the second internodes of WT and *cef3* plants. qRT-PCR analysis showed that the cellulose biosynthesis genes, such as *OsCESA4*, *OsCESA7*, *OsCESA9* and *BC12*, xylan biosynthesis genes, such as *OsCSLF6*, *OsIRX10*, *OsGT61-1*, *OsIRX8L* and *OsIRX14* were down-regulated in the *cef3* mutant, compared with WT (Fig. 8A, B). There is no significant difference in lignin biosynthesis genes expression between WT and *cef3* plants (Fig. 8C). Cellulose is synthesized by plasma membrane-localized cellulose synthase complexes (CSCs), which are assembled in either the endoplasmic reticulum (ER) or the Golgi apparatus and trafficked by vesicles to the plasma membrane (PM) [9]. To determine whether the CSCs trafficking are affected in *cef3* mutant, we examined the distribution and/or abundance of OsCESA9 between the PM and endomembrane systems in the second internodes of WT and *cef3* plants by western blotting. OsCESA9 is one isoform of the CSCs involved in cellulose biosynthesis of secondary cell walls. Given that there is no good endogenous OsCESA9 antibody in our lab, we first generated the transgenic plants that overexpress OsCESA9-Flag fusion protein in the *cef3* mutant background, and then cross into WT background. We further used anti-Flag antibodies to examine the subcellular distribution of OsCESA9-Flag, the western blot results showed that the level of OsCESA9-Flag has no significant difference in total membrane, but it was lower in the PEG (PM) and higher in the DEX (endomembrane) fractions (Fig. 9), respectively, compared with WT. Thus, CEF3, a protein involved in membrane trafficking, directly or indirectly affects SCW-related genes expression and CSCs abundance at the PM.

## Discussion

Secondary cell walls play a critical role in plant growth and development, and they also contain high amounts of lignocellulose, a key feedstock for the production of bio-energy and bio-based products [1]. Three major steps are necessary for conversion of lignocellulose to ethanol. i) physical and chemical pretreatments to enhance cell wall destruction, ii) enzymatic digestion to release soluble sugars, and iii) microbial fermentation to produce ethanol [37]. The first two of the three steps are mainly affected by lignocellulose recalcitrance of the secondary cell wall. Therefore, it is great significance for providing a strategy for manipulating plant biomass production to understand the mechanism underlying SCWs biosynthesis. So far, many brittle culm mutants have been isolated, and there are idea materials for studying SCWs biosynthesis [4, 29–31, 34–36]. In this study, we obtained a novel *culm easily fragile3* (*cef3*) mutant, which alters wall component (Table 1) and decreases the thickness of sclerenchyma cell walls (Fig. 2). These implying that *CEF3* may be involved in SCWs biosynthesis. In rice, OsCESA4, OsCESA7 and OsCESA9 comprise the CSCs necessary for SCWs biosynthesis [38]. All of these mutants show the brittle culm phenotype due to decreases in cellulose content [39–41]. We detected *OsCESA4*, *OsCESA7* and *OsCESA9* gene expression were downregulated in *cef3* plants (Fig. 8A), indicating that CEF3 is involved in SCWs biosynthesis may through regulating these gene expression. *CEF3* encodes a homologous protein of Arabidopsis STOMATAL CYTOKINESIS DEFECTIVE2 (SCD2), which together with SCD1 constitute the SCD complex that cooperates with members of the exocyst complex to mediate post-Golgi trafficking to the PM [22]. The subcellular localization results showed CEF3 is a Golgi-localized protein (Fig. 6B), and quantification of FM4-64 internalization assay revealed that CEF3 participated in membrane trafficking (Fig. 9), similar with its homolog of AtSCD2. We also found that the abundance of OsCESA9 was lower at the PM in *cef3* plants (Fig. 9). Therefore, as a critical protein involved in membrane trafficking, CEF3 may also contribute to the intracellular distribution of CSCs for regulating SCWs biosynthesis. BC3, a rice DRP OsDRP2B was also function in membrane trafficking pathways, its mutation also shows brittle culm phenotype and affects the distribution of proteins essential for cellulose biosynthesis in SCWs [13]. Not like *bc3* mutant, *cef3* exhibits brittle culm phenotype at after heading stage (Fig. 1) and no brittle culm phenotype at seedling stage (Additional file 1: Figure S1). Therefore, the biochemical and genetic relationships between BC3 and CEF3 in regulating secondary cell wall component trafficking in rice need further investigate. In the *cef3* plants, in addition to decreased cellulose content, other cell wall components were altered (Fig. 2H). The content of xylose, a major sugar of hemi-cellulose, was substantially decreased by approximately 18% (Table 1). We also detected some of xylan biosynthesis genes, such *OsIRX10*, *OsCSLF6*, *OsGT61-1*, *OsIRX8L* and *OsIRX14* gene expression were downregulated in *cef3* plants (Fig. 8B), indicating that CEF3 is involved in hemicellulose biosynthesis may through regulating these gene expression. The hemi-cellulose is produced in the Golgi apparatus, and then transported to the walls through secretory pathways [10]. Therefore, CEF3 not only affects the distribution of cellulose synthase at the PM, but also may affects the transport of hemicellulose from Golgi apparatus to the wall, and these hypotheses require further experimental verification.

In addition to the culm easily broken phenotype, *cef3* plant exhibits pleiotropic defects in plant height and panicle morphology (Fig. 1). We detected *CEF3* is ubiquitously expressed in all organs (Fig. 6A). This expression pattern also fits with the pleiotropy phenotypes (dwarfism, culm brittleness, small panicle etc*.*) of *cef3*. *BC12*, encodes a dual-targeting kinesin-4 protein, its mutation display dwarfism resulting from a significant reduction in cell number and brittleness due to an alteration in cellulose microfibril orientation and wall composition [33]. We detected decreased *BC12* expression level in *cef3* plants, suggesting CEF3 may regulate plant height development by regulating *BC12* gene expression.

As one of the most important staple food crops, rice produces significant quantities of agronomic biomass residue every year. In recent years, rice straw has been highlighted as an important material for biofuel production, but the high cellulose content and crystallinity determine lignocellulose recalcitrance, leading to costly biomass processing [31, 32]. Biomass enzymatic saccharification efficiency is a key parameter for determining lignocellulosic straw digestibility. In addition, we detected higher saccharification efficiency of the lignocellulosic material derived from the *cef3* mutants (Fig. 5 and Additional file 5: Figure S5), suggesting that the *CEF3* mutation can enhance biomass enzymatic saccharification. Therefore, we can used CRISPR/Cas9 system to edit the *CEF3* homologs in energy crops, such as poplar and switchgrass to improve biomass conversion efficiency.

## Conclusions

In this study, we isolated a novel culm easily broken mutant *cef3*, which exhibits altered cell wall composition and reduced secondary wall thickness. Map-based cloning revealed that *CEF3* encodes a homologous protein of Arabidopsis STOMATAL CYTOKINESIS DEFECTIVE2 (SCD2). Expression pattern analysis indicated that *CEF3* is ubiquitously expressed in many organs at different developmental stages. Subcellular localization revealed that CEF3 is a Golgi-localized protein. The FM4-64 uptake assay revealed CEF3 is involved in endocytosis. Furthermore, mutation of *CEF3* not only affected cellulose synthesis-related genes expression, but also altered the abundance of cellulose synthase catalytic subunit 9 (OsCESA9) in the PM and in the endomembrane systems. The saccharification assays revealed that CEF3 mutation can improve biomass enzymatic saccharification. Hence, this study has provided a powerful strategy for genetic modification of plant cell walls in bioenergy crops.

## Materials and methods

### Plant materials and growth conditions

The culm easily fragile (*cef3*) mutant was isolated from a *japonica* cultivar, xiushui63 (XS63) by heavy ion beam treatment. An F_2_ mapping population was generated from the cross between *cef3* and a polymorphic *indica* cultivar, 93–11. All plants used in this research were grown in the experimental fields at Hefei Institute of Physical Science, Chinese Academy of Sciences (Hefei, China) and Sanya (Hainan province, China) during the natural growing season.

### Measurement of extension force and microscopy

Extension force of the 2nd internodes and flag leaves of XS63, *cef3*, WYJ7 and *cef3-c1* plants were determined according to [29]. The maximum force required to break apart the internodes and leaves were considered as the extension strength of these plants at heading stage.

For transmission electron microscopy, the 2nd internodes of WT and *cef3* plants were cut with a razor and immediately post-fixed in 70% ethanol (V/V), 5% acetic acid (V/V), and 3.7% formaldehyde (V/V) mixture for at least 2 h. Samples were dried to the critical point, sputter-coated with gold, and observed with a scanning electron microscope (S570; Hitachi, Tokyo, Japan). The thickness of sclerenchyma cell wall was measured by Image J software.

### Cell wall composition analysis and saccharification assays

The second internodes of XS63, *cef3*, WYJ7, *cef3-c1* plants at mature stage were used to prepare alcohol-insoluble residues (AIRs) of the cell walls. De-starched AIRs were produced as previously described [35]. The samples were hydrolyzed in 67% v/v H_2_SO_4_ for 1 h at room temperature, and then in 2 M H_2_SO_4_ at 121 °C for 1 h (h). The alditol acetate derivatives were prepared from AIRs and then determined by GC–MS. The crystalline cellulose was measured with a modified Updegraff method, as previously described [42].

For saccharification assay, 1 mg (mg) destarched wall material was treated by boiled water for inactivating endogenous enzymes. After cooling down, the digestion was performed according to. The released sugars in the supernatant were measured by reading the A_540_ on an ELISA reader (Tecan) as described previously [43].

### Map-based cloning

The *cef3* locus was located and cloned using 1438 mutant F_2_ plants, which were selected from the population of *cef3* and 93–11. Molecular markers distributed throughout the whole rice genome were used for *cef3* locus rough mapping. Molecular markers for fine mapping were developed to narrow the *cef3* locus to a 52 kb region on chromosome 1. The DNA fragments that correspond to the 7 ORFs in this mapping region were amplified from *cef3* and WT plants using KOD DNA polymerase (TOYOBO, http://www.toyobo.co.jp/e/bio) and Sequenced by BioSune company (http://www.biosune.com). For complementation of *cef3* mutant, the full-length *CEF3* coding sequence, a 2.5-kb fragment of upstream sequence from ATG and 0.2 kb fragment of downstream sequence from TGA were inserted into binary vector pCAMBIA2300 between the EcoRI and BamHI sites to generate the construct *pCEF3::CEF3* (*pCEF3F*), which was introduced into *cef3* plants by the *Agrobacterium*-mediated transformation procedure as described previously [7]. The primers were used in map-based cloning are listed in Additional file 8.

### Expression analysis

Total RNA was extracted from various rice tissues using TRIzol reagent (Invitrogen), as described previously [4]. The complementary DNA (cDNA) was synthesized from total RNA using a reverse transcriptional kit (TransGen, http://www.transgen.com.cn/). Quantitative RT-PCR was performed using relevant primers and qRT-PCR kit (TransGen, http://www.transgen.com.cn/) on a quantitative 7500 PCR system (ABI). All assays were repeated at least 3 times, the *OsActin2* gene was used as an internal control. The primers were used in expression analysis are listed in Additional file 8.

### Subcellular localization of CEF3

To observe the subcellular localization of CEF3, a green fluorescent protein (GFP) fused to the C-terminus of CEF3 and inserted into the *pCAMBIA1300* between the *Kpn*Ι and *BamH*Ι sites to create the *p35S::CEF3-GFP* vector, which was introduced into *A. tumefaciens* GV3101 and transformed into 1-month-old *N. benthamiana* leaves together with a vector harboring the cis-Golgi marker Man49-mCherry. The fluorescent signals were observed with a confocal laser scanning microscope (Leica TCS SP5) after 3 days.

### Binary vectors construction and rice transformation

We used CRISPR/Cas9 system for creating *cef3* mutants. The CRISPR/Cas9 binary vectors were constructed as previously described [44]. The Cas9 plant expression vector (*pYLCRISPR/Cas9Pubi-H*) and sgRNA expression vector (*pYLgRNA*) were provided by Prof. Yao-Guang Liu (South China Agricultural University). We selected the target (TCAAAACCAGCACGACATTG) in the 2nd exon of *CEF3* (Fig. 4a) as candidate target sequences according to the design principles of the target sequences in the CRISPR/Cas9 system. Then, they were ligated into two sgRNA expression cassettes of a Cas9 binary vector, driven by *OsU3* promoter. The construct was introduced into a *japonica* cultivar, wuyunjing7 (WYJ7) by the *Agrobacterium*-mediated transformation procedure as described previously [7].

### Western blot

The extraction of total membrane protein and separation of the plasma membrane (PM) and endomembrane fractions were performed as previously described [41]. The proteins in the PEG and DEX fractions were separately collected and concentrated at 100,000 g for 1 h. The pellets were dissolved in suspension buffer (2 mM Tris, pH 6.5, 1 mM DTT and 0.25 M sucrose). Ten micrograms of protein was run on an SDS–PAGE gel and probed with corresponding monoclonal antibodies and the secondary antibody HRP-conjugated anti-mouse IgG (Sigma). The reactions were determined by the ECL Plus Western Blotting Detection System kit (GE Healthcare) and the chemiluminescent signal intensity was detected with a Tanon-5200 Chemiluminescent Imaging System (Tanon Science and Technology). The antibodies of Anti-Flag, Anti-HSP90, Anti-PIP1s and Anti-BiP2 were purchased from Agrisera (http://www.agrisera.com).

## Supplementary Information

**Additional file 1**: **Figure S1**. Phenotype of wild-type (WT), *cef3 *and *cesa9* plants at seedling stage. The seedling of *cesa9* is easily broken, while seedlings of WT and *cef3* are normal after hand flexing

**Additional file 2**: **Figure S2**. CEF3 amino acid sequence alignment of WT and *cef3* mutant

**Additional file 3**: **Figure S3**. Complementary assay. The phenotype (A) and the plant height (B) show the rescued properties in the complemented plants. Scale bars: 10 cm.

**Additional file 4**: **Figure S4**. Deduced CEF3 amino acid sequence alignments for the three homozygous mutants generated by CRISPR-Cas9 system and WT.

**Additional file 5**: **Figure S5**. Saccharification analysis of the wall residues from WT and *cef3-c1 *internodes. The wall residues were treated with enzyme mixture for 5 and 20 h. Error bars indicate SE from the mean of three replicates. Different letters denote significant differences (P < 0.05, Duncan’s multiple range test).

**Additional file 6**: **Figure S6**. Phenotypic comparison among the wild-type and *cef3 *plants generated by CRISPR–Cas9 system. (A) The phenotype of the wild-type (WT) and *cef3* mutants generated by CRISPR-Cas9 system at the mature stage. Scale bars: 10 cm. (B) Folding the internodes of the wild-type,* cef3-c2* and *cef3-c3*. Scale bars: 2 cm (C) The plant height of the wild-type, *cef3-c2 *and *cef3-c3*. (D) The extension force of culms in the wide-type, *cef3-c2 *and *cef3-c3 *plants. Error bars represent SE (n = 30). Different letters denote significant differences (P < 0.05, Duncan’s multiple range test)

**Additional file 7**: **Figure S7**. (A) Phylogenetic tree of CEF3 in rice and Arabidopsis. (B) The amino acid sequence alignment between the AtSCD2 and OsCEF3. Different rectangles represent different functional domains.

**Additional file 8**: The primers used for DNA constructs and Real-time analysis in this study.

**Additional file 9**: **Table S1** Cell wall composition analysis of internodes of wild type and *cef3*-*c1* plants.

## Data Availability

All data supporting the conclusions of this article are provided within the article and its Additional files 1: Figure S1, Additional files 2: S2, Additional files 3: S3, Additional files 4: S4, Additional files 5: S5, Additional files 6: S6, Additional files 7: S7, Additional files 9: Table S1, and Supplementary data-primers were used in this study).
