# Supplementary figures and images for: CEF3 is involved in membrane trafficking and essential for secondary cell wall biosynthesis and its mutation enhanced biomass enzymatic saccharification in rice
Source: Biotechnol Biofuels Bioprod. 2022 Oct 14;15:111. doi: 10.1186/s13068-022-02205-y (PMC9569061; doi:10.1186/s13068-022-02205-y)

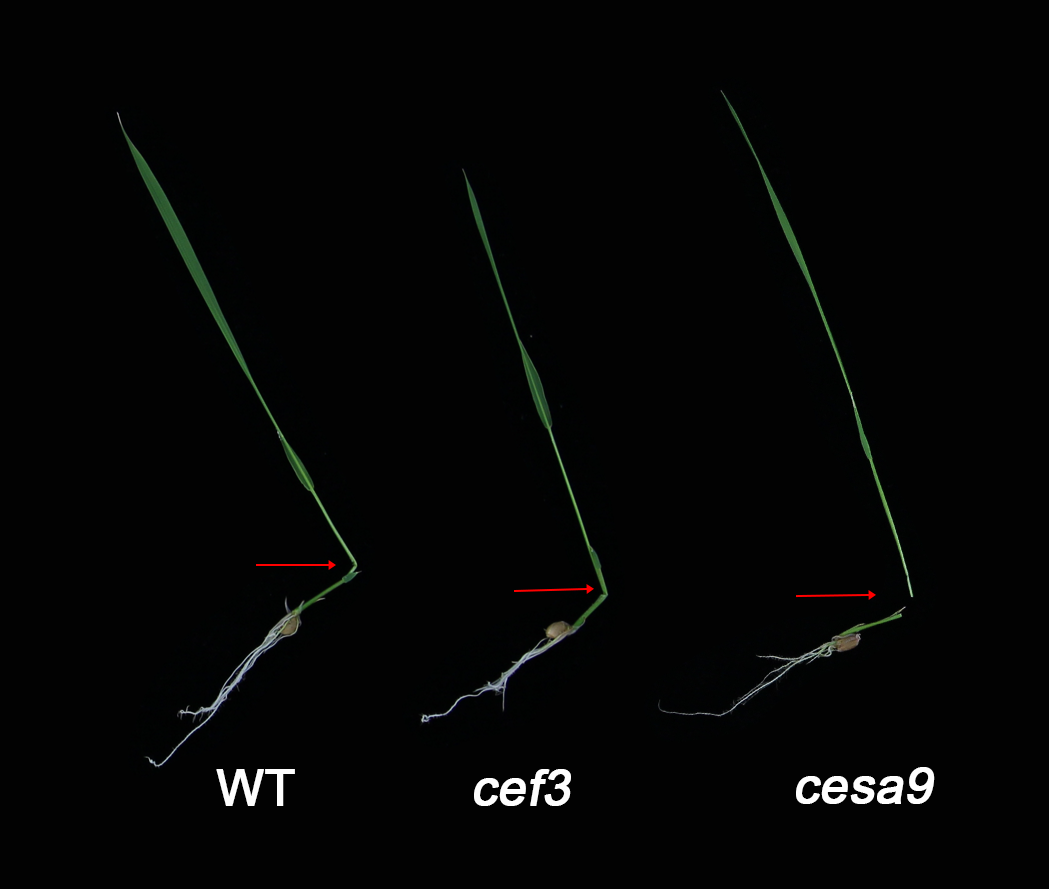

Supplement: Supplementary file 1 — Additional file 1: Figure S1. Phenotype of wild-type (WT), cef3 and cesa9 plants at seedling stage. The seedling of cesa9 is easily broken, while seedlings of WT and cef3 are normal after hand flexing [file 13068_2022_2205_MOESM1_ESM.tif]

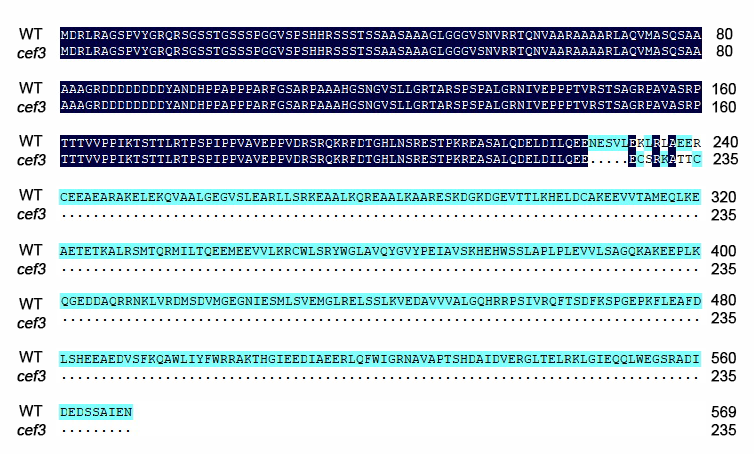

Supplement: Supplementary file 2 — Additional file 2: Figure S2. CEF3 amino acid sequence alignment of WT and cef3 mutant [file 13068_2022_2205_MOESM2_ESM.tif]

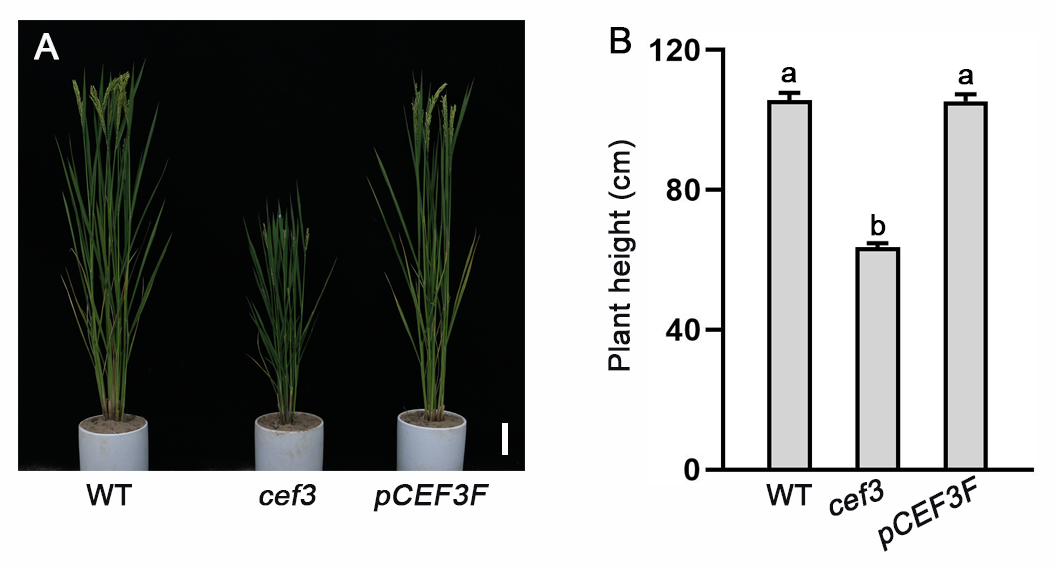

Supplement: Supplementary file 3 — Additional file 3: Figure S3. Complementary assay. The phenotype (A) and the plant height (B) show the rescued properties in the complemented plants. Scale bars: 10 cm. [file 13068_2022_2205_MOESM3_ESM.tif]

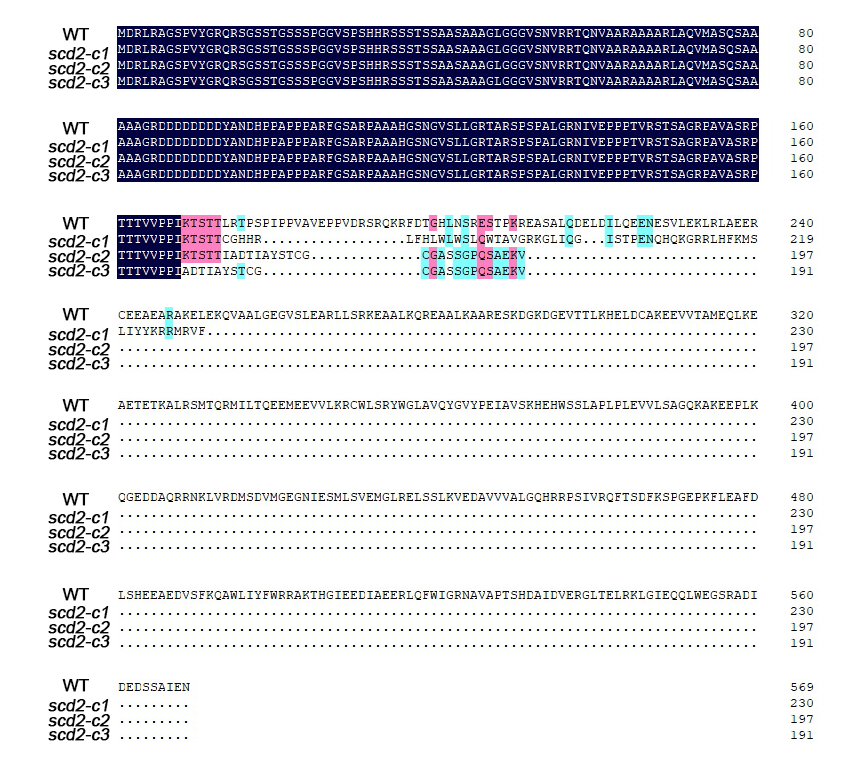

Supplement: Supplementary file 4 — Additional file 4: Figure S4. Deduced CEF3 amino acid sequence alignments for the three homozygous mutants generated by CRISPR-Cas9 system and WT. [file 13068_2022_2205_MOESM4_ESM.tif]

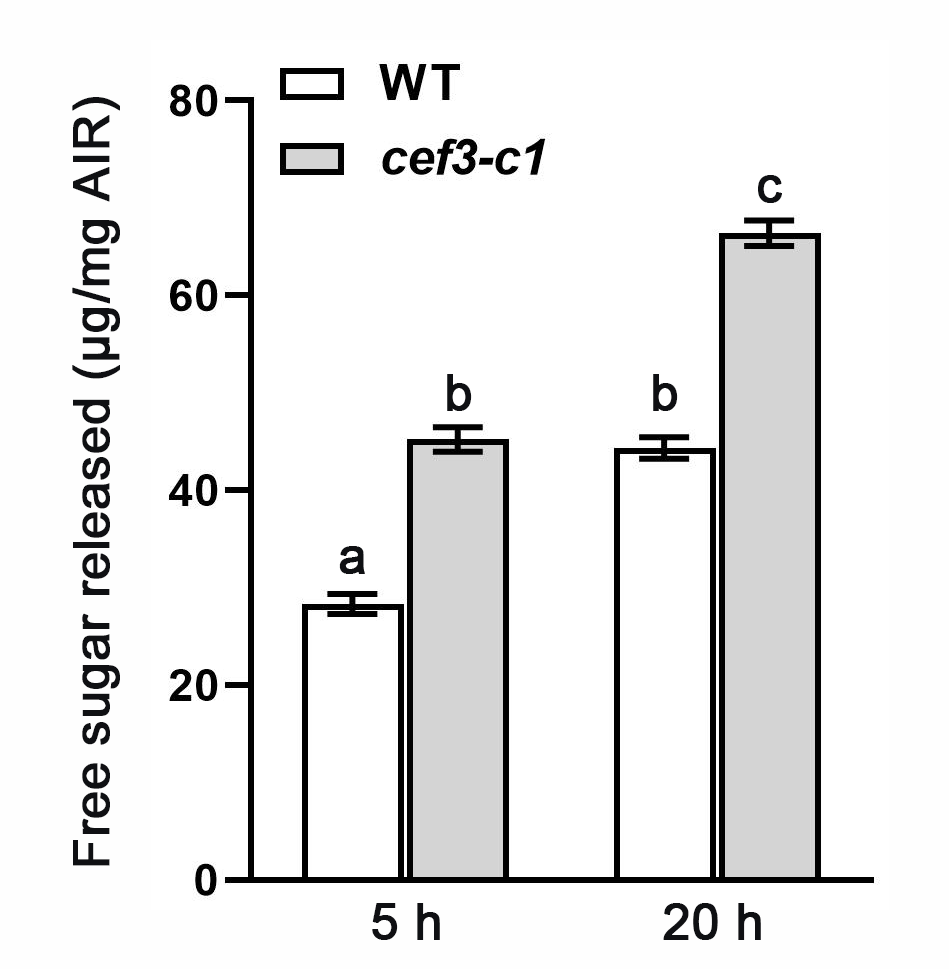

Supplement: Supplementary file 5 — Additional file 5: Figure S5. Saccharification analysis of the wall residues from WT and cef3-c1 internodes. The wall residues were treated with enzyme mixture for 5 and 20 h. Error bars indicate SE from the mean of three replicates. Different letters denote significant differences (P < 0.05, Duncan’s multiple range test). [file 13068_2022_2205_MOESM5_ESM.tif]

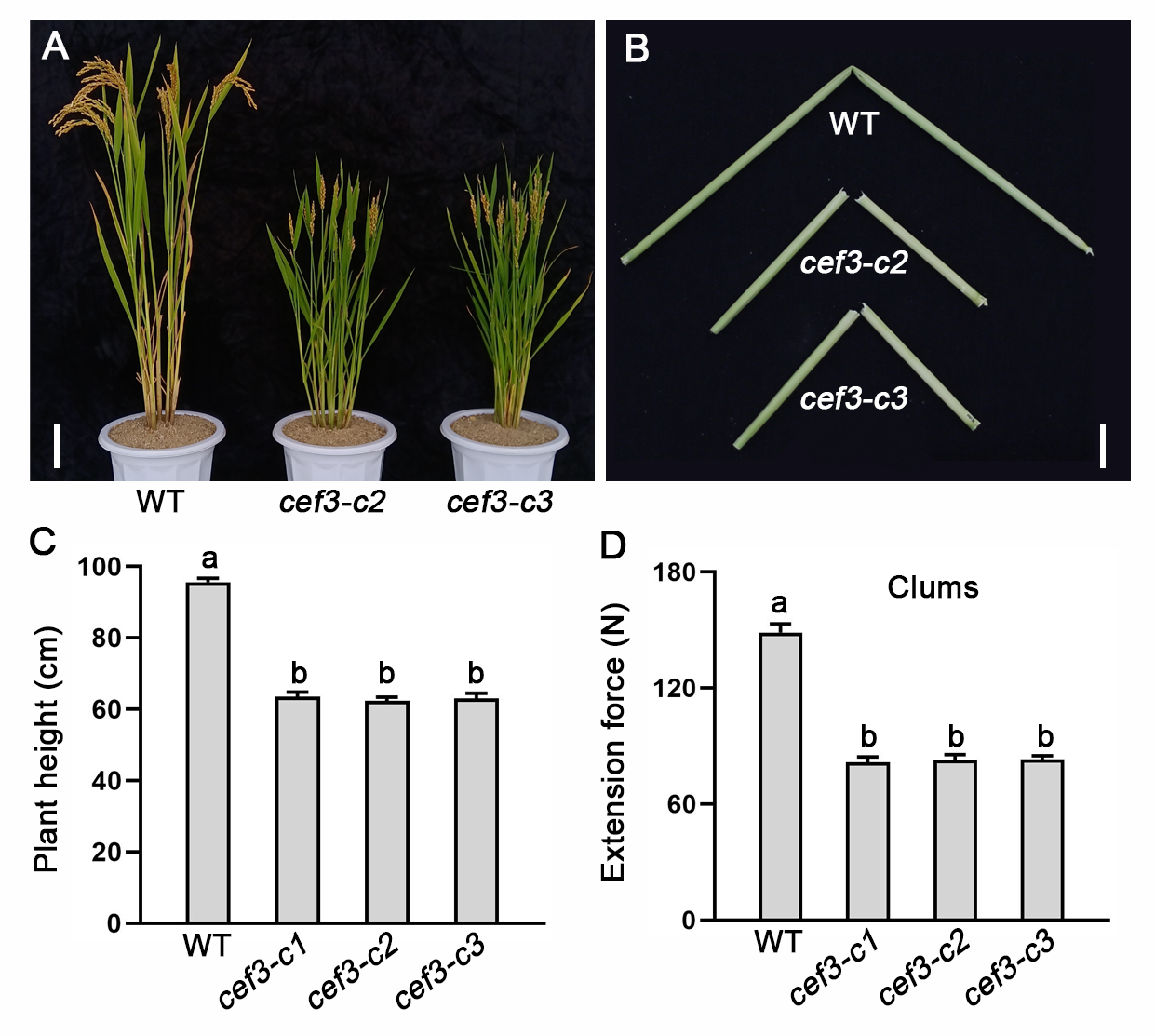

Supplement: Supplementary file 6 — Additional file 6: Figure S6. Phenotypic comparison among the wild-type and cef3 plants generated by CRISPR–Cas9 system. (A) The phenotype of the wild-type (WT) and cef3 mutants generated by CRISPR-Cas9 system at the mature stage. Scale bars: 10 cm. (B) Folding the internodes of the wild-type, cef3-c2 and cef3-c3. Scale bars: 2 cm (C) The plant height of the wild-type, cef3-c2 and cef3-c3. (D) The extension force of culms in the wide-type, cef3-c2 and cef3-c3 plants. Error bars represent SE (n = 30). Different letters denote significant differences (P < 0.05, Duncan’s multiple range test) [file 13068_2022_2205_MOESM6_ESM.tif]

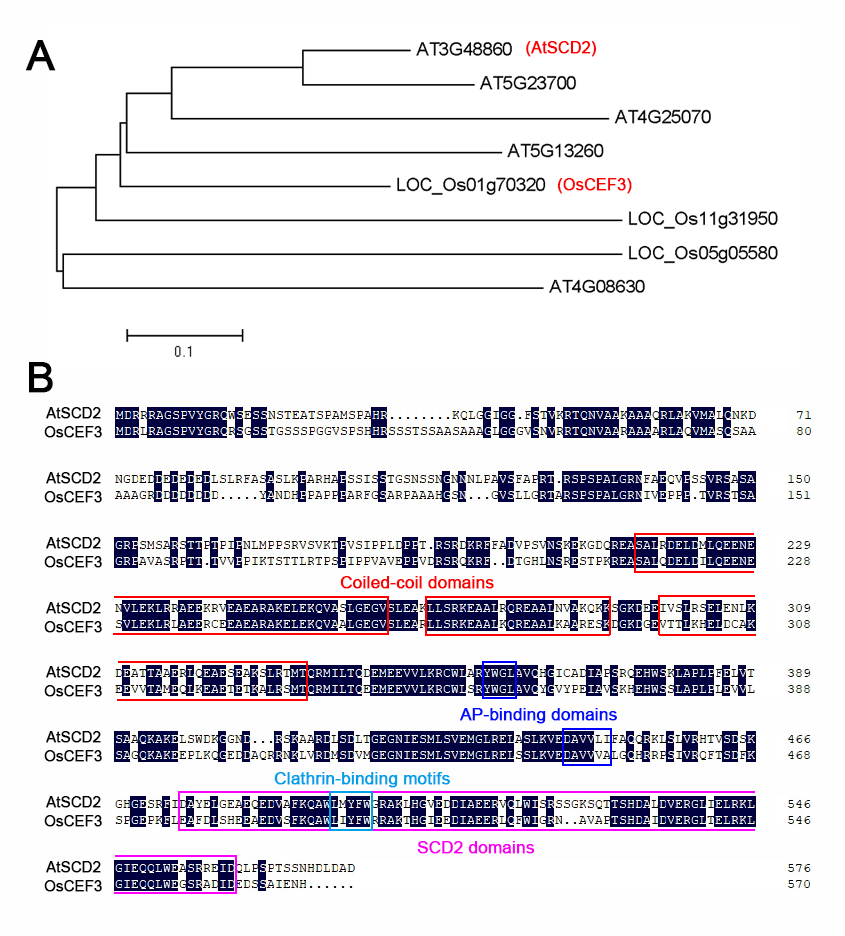

Supplement: Supplementary file 7 — Additional file 7: Figure S7. (A) Phylogenetic tree of CEF3 in rice and Arabidopsis. (B) The amino acid sequence alignment between the AtSCD2 and OsCEF3. Different rectangles represent different functional domains. [file 13068_2022_2205_MOESM7_ESM.tif]
