# Supplementary material for: CEF3 is involved in membrane trafficking and essential for secondary cell wall biosynthesis and its mutation enhanced biomass enzymatic saccharification in rice
Source: Biotechnol Biofuels Bioprod. 2022 Oct 14;15:111. doi: 10.1186/s13068-022-02205-y (PMC9569061; doi:10.1186/s13068-022-02205-y)
Supplement: Supplementary file 9 — Additional file 9: Table S1 Cell wall composition analysis of internodes of wild type and cef3-c1 plants. [file 13068_2022_2205_MOESM9_ESM.docx]

TableS1 Cell wall composition analysis of internodes of wild type and *cef3*-*c1* plants

| Sample | Rha | Fuc | Ara | Xyl | Man | Gal | Glu | Cellulose |
| --- | --- | --- | --- | --- | --- | --- | --- | --- |
| Wild type | 1.65±0.08 | 0.56±0.05 | 17.72±1.25 | 124.21±4.64 | 1.33±0.10 | 6.54±1.42 | 64.40±8.42 | 474.52±13.60 |
| *cef3-c1* | 2.13±0.04* | 0.84±0.01 | 25.44±0.48* | 84.01±6.68* | 1.37±0.04 | 22.21±0.28* | 168.81±3.74* | 368.30±12.16* |

The results are means ±SE of five independent assays. Each wall component was calculated as mg·g^-1^ of alcohol-insoluble cell-wall residue.

*Significant difference (*t*-test at P < 0.01) with respect to wild-type.
